# Caffeine in the Diet: Country-Level Consumption and Guidelines

**DOI:** 10.3390/nu10111772

**Published:** 2018-11-15

**Authors:** Celine Marie Reyes, Marilyn C. Cornelis

**Affiliations:** Department of Preventive Medicine, Northwestern University Feinberg School of Medicine, Chicago, IL 60611, USA; celine.reyes@northwestern.edu

**Keywords:** caffeine, coffee, tea, soda, energy drinks, mate, guidelines, country, consumption, population, public policy

## Abstract

Coffee, tea, caffeinated soda, and energy drinks are important sources of caffeine in the diet but each present with other unique nutritional properties. We review how our increased knowledge and concern with regard to caffeine in the diet and its impact on human health has been translated into food-based dietary guidelines (FBDG). Using the Food and Agriculture Organization list of 90 countries with FBDG as a starting point, we found reference to caffeine or caffeine-containing beverages (CCB) in 81 FBDG and CCB consumption data (volume sales) for 56 of these countries. Tea and soda are the leading CCB sold in African and Asian/Pacific countries while coffee and soda are preferred in Europe, North America, Latin America, and the Caribbean. Key themes observed across FBDG include (i) caffeine-intake upper limits to avoid risks, (ii) CCB as replacements for plain water, (iii) CCB as added-sugar sources, and (iv) health benefits of CCB consumption. In summary, FBDG provide an unfavorable view of CCB by noting their potential adverse/unknown effects on special populations and their high sugar content, as well as their diuretic, psycho-stimulating, and nutrient inhibitory properties. Few FBDG balanced these messages with recent data supporting potential benefits of specific beverage types.

## 1. Introduction

Caffeine is the most widely consumed psychostimulant in the world [1]. It occurs naturally in coffee beans, tea leaves, cocoa beans, and kola nuts, and is also added to foods and beverages. Important dietary sources include coffee, tea, yerba mate, caffeinated soda (cola-type), and energy drinks [2]. There is increasing public and scientific interest in the potential health consequences of habitual intake of these caffeine-containing beverages (CCB). Rigorous reviews of caffeine toxicity conclude that consumption of up to 400 mg caffeine/day in healthy adults is not associated with adverse effects [3,4,5]. Epidemiological studies support a beneficial role of moderate coffee intake in reducing risk of several chronic diseases, but heavy intake is likely harmful regarding pregnancy outcomes [6]. Health implications of regular tea, mate, and energy drink consumption are inconclusive and most concern for caffeinated soda intake currently pertains to its sugar content and relationship to obesity [7,8,9,10,11,12].

CCB also contribute a wealth of other compounds to the diet that have potential benefits or risks to health and thus it is imperative to consider the context (i.e., beverage type) in which caffeine is consumed [12,13,14,15,16]. Food-based dietary guidelines (FBDG) provide context-specific advice on healthy diets that are evidence-based and respond to a country’s public health and nutrition priorities, sociocultural influences, and food production and consumption patterns, among other factors [17]. These factors change over time, and in turn, so do FBDG. Our knowledge and concern with regard to caffeine sources in the diet and their impact on human health has increased over the years. We therefore sought to review how such knowledge and concern has been translated into FBDGs and within the context of what each country actually consumes.

## 2. Material and Methods

### 2.1. Data Collection Strategy for Dietary Caffeine Guidelines

Figure S1 outlines our data collection strategy. We initially used the Food and Agriculture Organization (FAO) website, which provided general food-based dietary guidelines (FBDG) from each country (http://www.fao.org/nutrition/nutrition-education/food-dietary-guidelines/en/). Each country’s page included the most recent publication date of the guidelines, intended audience, general FBDG messages, downloadable guidelines if available, and contact information of those governmental institutions that established the guidelines.

The general messages from FAO were the first resource for any guidelines pertaining to caffeine or CCB including coffee, tea, yerba mate, energy drinks, and carbonated soft drinks. Beverages were a focus of the current review because they are the primary contributors of caffeine in the diet [2,18]. Other caffeine sources, such as products containing cocoa and kola nut, contribute relatively small amounts to the diet [18]. We considered guidelines for the broader categories of soft drinks or sugar-sweetened beverages (SSB) since colas (typically containing caffeine) were rarely distinguished from these other beverages. Non-caffeine-containing teas were also considered because some FBDG provide different guideline for these teas and regular tea (i.e., black or green tea) that might provide additional insight into the underlying reason for the recommendations. We then accessed the downloadable materials if available to search for more caffeine-related messages. For materials published in foreign languages, we found translators using the Cochrane Task Exchange or personal contacts. Additionally, we used the contact information from the FAO page to inquire via email or web applications about any updated or additional caffeine-related guidelines that were not available via the FAO website. Finally, after these search efforts, for countries with limited or no information regarding dietary caffeine, we searched for publications related to national dietary guidelines and contacted the authors for further information.

Countries were classified according to the World Bank income classification [19]. We also used the non-comprehensive World Cancer Research Fund International NOURISHING database to identify actions in place by countries that attempt to regulate dietary caffeine consumption [20,21].

### 2.2. Data Resource for Dietary Caffeine Consumption

We adopted country-level volume sales of CCB as a proxy measure of CCB consumption and these were estimated using the Euromonitor Passport Global Market Information Database [22]. Euromonitor collects these data from trade associations, industry bodies, business press, company financial reports, and official government statistics. Specifically, we downloaded (bulk format) 2017 country-specific annual sales of (i) coffee, total brewed volume (liters); (ii) tea, total brewed volume (liters); (iii) “other hot drinks,” total brewed volume (liters); (iv) carbonates, total volume (liters); (v) sports and energy drinks, total volume (liters); (vi) ready-to-drink (RTD) coffee, total volume (liters); and vii) RTD tea, total volume (liters). For each country, data for each beverage was presented as a proportion of total CCB volume sales. Total CCB volume sales were also expressed on a per capita basis using total population estimates for 2017 (also downloaded from Euromonitor). For each country, we additionally reviewed the 2017 detailed report to collect information on the most common type or category of each beverage sold. Additional details for “sports and energy drinks,” RTD coffee and RTD tea were not systematically collected as they were not uniformly available across countries. Aside from including yerba mate, the “other hot drinks” category was deemed an unlikely key source of CCB and thus we only make reference to this category as appropriate.

## 3. Results

**Figure 1:**
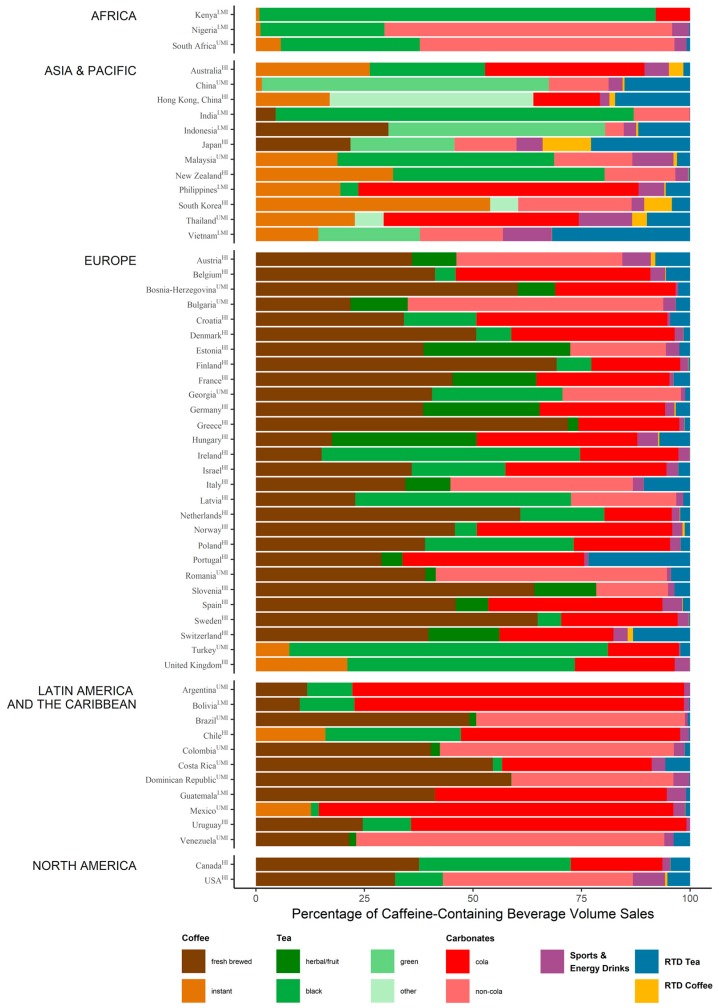
Percentage of caffeine-containing beverage volume sales per beverage (Euromonitor 2017). Subcategories of coffee, tea, and carbonates were assigned according to the most commonly, but not exclusively, consumed beverage type in that category. Countries were classified by income based on World Bank 2017. HI: high-income; LI: low-income; LMI: lower-middle-income; UMI: upper-middle-income. Data for RTD beverages were incomplete for Kenya, Nigeria, South Africa, India, Bosnia-Herzegovina, Croatia, Estonia, Georgia, Latvia, Slovenia, Bolivia, Costa Rica, Dominican Republic, Guatemala, Uruguay, and Venezuela.

Using the FAO listing of the most recent FBDG from 90 countries as a starting point, we found any mentions of caffeine or CCB in 81 of these, which are summarized in Table 1. Sixty-six of these were published in the last ten years. The oldest guidelines were published by Venezuela (1991) and Greece (1999). Intended audiences for each FBDG are provided in Table S1. Most FBDG were intended for the general, healthy population over 2 years of age with several FBDG including specific guidelines for subgroups of the population such as children and pregnant/nursing mothers. Euromonitor annual volume sales of CCB in 2017 were available for 56 of the 90 FAO countries. Euromonitor data was not available for countries of the Near East (as defined by FAO). Figure 1 presents the percentage of caffeine-containing beverage volume sales per beverage per country. Subcategories of coffee, tea, and carbonates were assigned according to the most commonly, but not exclusively, consumed beverage type in that category. North America (defined by FAO as including Canada and USA) had the highest average country annual total CCB volume sales per capita (348 L/capita), followed by Europe (200 L/capita), Latin America and the Caribbean (153 L/capita), Asia and the Pacific (126 L/capita), and Africa (90 L/capita).

### 3.1. Africa

The most commonly consumed CCB in African countries include tea and carbonated soda. Tea is typically of the black type while carbonated drinks are commonly non-cola-type (unlikely caffeinated including lime, ginger ale, tonic water, orange carbonates, and “other”). When coffee is consumed, it is usually of the regular (not decaffeinated, >95% of sales) and instant type. Data for RTD coffee/tea were not complete for these selected African countries.

Five African countries have published FBDG that consider dietary caffeine sources in some context. Nigeria, Sierra Leone, and South Africa discourage high coffee and tea intake because they inhibit iron bioavailability or increase phosphorous levels. South Africa’s guidelines for ages 5+ nevertheless support the intake of these beverages as a means to attain adequate fluid intake, further noting that any diuretic effects of caffeine are only a concern for individuals unaccustomed to regular caffeine intake. Most FBDG discourage caffeinated soda, but only due to their high sugar content.

### 3.2. Asia and the Pacific

Tea and carbonated soda are the leading CCB sold across Asia and the Pacific countries. High carbonated soda consuming countries prefer the cola type, while low consuming countries prefer non-colas. Black, green, and “other” teas are major tea types consumed. RTD teas are also popular in Japan, Hong Kong, and Vietnam. Most coffee that is consumed is of the regular (>97% of sales) and instant type; instant mixes (coffee, sugar, and cream powder) are especially popular in South Korea, Hong Kong, Malaysia, Thailand, Philippines, Vietnam, and China.

Six of the fifteen included countries of Asia and the Pacific express caution concerning the iron inhibitory effects of coffee and tea, particularly when these beverages are consumed with meals. China, India, Indonesia, New Zealand, and Korea all advise pregnant and lactating women to minimize their intake of CCB. Indonesia and New Zealand further cite research supporting caffeine limits of 250–300 mg/day for these women. Potential diuretic effects of caffeine are discussed in guidelines for Fiji, Indonesia, and Malaysia. According to Fiji and Indonesia, heavy tea and coffee consumers may need to adjust their water intake, while Malaysian guidelines note little concern regarding the diuretics effects of CCB in amounts typically consumed. India’s guidelines discuss the stimulant effects of caffeine present in coffee and tea and advise moderation when consuming these beverages. Excess consumption of coffee was viewed unfavorably for cardiovascular health, while any potential benefits noted for tea consumption were off-set by its caffeine content. The majority of FBDG discouraged caffeinated soda due to its high sugar content. New Zealand further referenced the caffeine content of these beverages, discouraging the intake of these and other caffeine-containing beverages among children and adolescents. With some concern of caffeine’s impact on bone health, older people in New Zealand are advised to consume no more than 300 mg of caffeine per day. Moderate amounts of tea and coffee are also advised for adults; advice that aims to balance the beneficial and potentially adverse properties of these beverages attributable to polyphenol, caffeine, and tannin content. Sri Lanka also noted that tea without milk and sugar has some antioxidants that benefit health.

### 3.3. Near East

FBDG for Iran advise the general population to reduce soft drink consumption in the context of reducing overall sugar intake. Lebanon’s guidelines advise individuals to avoid consuming coffee, tea, or caffeinated sodas with meals as they inhibit dietary iron absorption. Despite notes concerning caffeine’s diuretic effects, tea and coffee are the preferred beverages (after water) for hydration. Sweetened beverage intake should be limited according to Qatar’s guidelines and in this context, soda and energy drinks are discouraged and careful attention made to the amount of sugar added to coffee.

### 3.4. Europe

Overall, coffee and carbonated soft drinks are the top CCB sold in Europe. Netherlands consumes the largest volume of coffee per capita than any other country in the Euromonitor database, followed by Finland and Sweden. U.K. and Turkey prefer instant coffee while the rest of Europe prefers fresh-brewed coffee. Decaffeinated coffee accounts for ≈8% of coffee sold in Spain and U.K. and <5% for other parts of Europe. Most carbonated soft drinks sold are of the cola-type. Ireland, Turkey, U.K., and Latvia prefer tea over other CCB. Ireland consumes the more tea per capita than any other country in the Euromonitor database. Black and fruit/herbal teas are the most commonly consumed teas across Europe. Sports and energy drinks and RTD teas are consumed at varying amounts across Europe while RTD coffee consumption is uncommon.

Thirty European countries have published FBDG that consider dietary caffeine sources in some context. Albania, Georgia, Latvia, and Romania are the only set of guidelines noting the iron inhibitory effects of coffee and tea. Latvia and Croatia’s guidelines stated that coffee and tea can reduce calcium absorption. Albania, Latvia, and Portugal were the only FBDG that discouraged the intake of CCB to meet daily water requirements. Most FBDG that specifically mention limiting soda or the broader SSB category do so in the context of limiting sugar intake. Energy drinks are discouraged in Malta guidelines due to their sugar as well as stimulant content. Albania, Belgium, Denmark, Ireland, Latvia, and Romania advise pregnant and lactating women to minimize consumption of coffee, tea, or other CCB (≤200–300 mg caffeine/day). Albania, Denmark, Hungary, and Portugal discourage caffeine intake among children. In Denmark and the U.K., adults are advised to limit caffeine intake to 400 mg/day. In Portugal, this limit is set to 300 mg/day. Netherlands’ guidelines recommend the daily consumption of three cups of green or black tea on the basis of research showing it reduces risk of stroke, blood pressure, and possibly diabetes. Similar benefits are stated for coffee consumption, but the Dutch are only advised to replace unfiltered coffee with filtered coffee due to known cholesterol-raising substances present in the former. Romania notes that tea is an important source of bioflavonoids with antioxidant properties that might protect against cardiovascular disease (CVD) but does not provide recommendations for tea per se. In contrast, Latvia discourages the use of coffee or tea in place of water or herbal teas for hydration, in part, for mental health and heart disease prevention.

### 3.5. Latin America and the Caribbean

Carbonated soda (mostly cola-type) and coffee (mostly fresh-brewed) are the most commonly sold CCB in Latin America and the Caribbean. Argentina and Uruguay are also heavy consumers of yerba mate [22]. Uruguay has the highest per capita consumption of yerba mate in the world [22,23,24]. Other CCB are less commonly consumed across this region compared to other regions of the world.

Twenty-six countries of Latin America and the Caribbean have published FBDG with some mention of dietary caffeine. All countries advised limiting SSB (including soda). Only seven guidelines made specific reference to coffee, tea or caffeine. Yerba mate was not specifically mentioned in any FBDG. Pregnant and lactating mothers in Chile are advised to limit tea and coffee intake while Colombian guidelines advise they avoid energy drinks. In FBDG of Bolivia, Guatemala and Honduras, coffee, tea, and caffeine more generally, were discouraged as substitutes for water because they are diuretics, acidic and/or lead to digestive system problems. In Mexican guidelines, non-sweetened coffee and tea are limited to four cups/day. In Brazil, unsweetened coffee and tea were acceptable substitutes for water.

### 3.6. North America

In North America, fresh-brewed coffee and carbonated sodas are the most commonly sold CCB. Tea (mostly black) and other CCB are common as well. The USA consumed the most carbonated soda and sports and energy drinks per capita than any other country in the Euromonitor database.

Canadian and American guidelines for CCB were based on evidence compiled and reviewed, in part, for the purpose of setting national guidelines [4,25]. Canada’s FBDG include caffeine upper limits ranging from 45 to 85 mg/day for ages 4 through 12 years, 2.5 mg/kg body weight for adolescents aged 13+, 400 mg/day for adults, and 300 mg/day for pregnant or breastfeeding women, as well as women planning to become pregnant. In the USA, three to five cups of coffee/day (providing up to ≈400 mg/day caffeine) is considered safe for adults, yet individuals who do not consume regular coffee or other caffeinated beverages are not encouraged to begin doing so. Pregnant and breastfeeding women are encouraged to consult their health care providers for advice concerning caffeine intake. Sodas and energy drinks are discouraged but more with regard to their sugar content. Caution is also advised when mixing caffeine and alcohol.

## 4. Discussion and Conclusions

The goal of the current review was to provide the first world summary of guidelines pertaining to dietary caffeine consumption. CCB, while major contributors to caffeine in the diet, also present with other unique nutritional properties. We therefore leveraged existing FBDG since they emphasize food-specific rather than nutrient-specific advice on healthy diets and are developed by interdisciplinary teams of experts with many sources of information reviewed in the process [17]. We begin our discussion with country differences in consumption habits that extend the macro-level consumption data we present in the current report. These are followed by key themes observed across country FBDG including (i) caffeine-intake upper limits to avoid potential health risks, (ii) CCB as replacements for plain water, (iii) CCB as added-sugar sources, and (iv) health benefits of caffeine-containing beverage consumption.

Consumption habits are greatly affected by factors such as geographical origin, culture, lifestyle, social behavior, and economic status. Although regular coffee dominates over decaffeinated coffee across countries, coffee brewing methods differ and these are only partly captured by Euromonitor data used in the current report. While drip filter coffee is the most popular brewing method worldwide, plunger coffee dominates in northern Europe. Turkish coffee is popular in the Middle East, Greece, Turkey, and Eastern Europe, and Espresso and Moka methods are the most common in Italy, Spain, and Portugal [26,27,28,29,30,31,32,33,34,35,36,37]. Tea habits also vary around the world [38,39,40]. For example, Western countries generally drink black tea, made by pouring boiling water over a teabag in a pot or mug and allowing it to steep before consuming (either with or without milk and/or sugar). In India, Pakistan, and some Middle Eastern countries, black tea is largely prepared by boiling the black leaves in a pan for several minutes prior to consumption (often together with water, milk, and sugar). In China and Japan, the drink is normally prepared from green tea by infusing it in hot (but not boiling) water and only the second and subsequent infusions are consumed [40]. Yerba mate is consumed in several South American countries, where it originated, but is less common to other parts of the world [12,22]. Grounded and dried yerba mate leaves and stems are widely consumed in the form of infusions, such as chimarrão and tererê, prepared with hot and cold water, respectively [15]. Differences in brewing methods as well as the type and processing of beans/leaves/stems used are relevant since all affect the sensorial quality and the amount and type of compounds in a “cup” of coffee, tea, or mate [12,15,41]. For example, one needs to consume about three Turkish and five Espresso coffee cups to acquire the same amount of caffeine in one American cup [41]; details to consider when comparing country guidelines. In contrast to the aforementioned natural sources of caffeine, there is little evidence, to our knowledge, in support of a true cultural component to consumption of caffeine-added beverages such as caffeinated soda and energy drinks. A global and concerning pattern is that caffeine-added beverages, which have potential health risks and no benefits, are the primary contributors to caffeine in the diet of children and adolescents [18,42,43,44,45,46,47,48,49,50,51,52].

FBDG respond to a country’s public health and nutrition priorities, sociocultural influences and food production and consumption patterns, among other factors [17]. Historically, the FBDG have focused on undernutrition and included guidelines aimed at consuming a diverse diet to address energy and nutrient gaps [53]. With time, many FBDG have evolved to include guidelines to support healthy lifestyle and specific recommendations to target various age groups [53]. In general, FBDG of some countries such as Sri Lanka, Sierra Leone, and Bangladesh address nutrient inadequacies in the population [54,55,56,57,58,59], while those of other countries, such as India, Thailand, Iran, Lebanon, and Brazil, address the double burden of undernutrition and overnutrition [54,60,61,62,63,64,65]. FBDG of developed and high-income countries, much of Europe and North America, are largely intended for prevention of chronic disease, adverse symptoms, or side effects [25,66,67]. These nutritional priorities partly determined if and how dietary caffeine sources were incorporated into guidelines.

For infants, children and adolescents, CCB consumption is often simply discouraged in FBDG. Canada is an exception and provides quantitative upper limits for caffeine intake according to age. For some countries, such as Nigeria, South Africa, Greece, and Mexico, it is not uncommon to introduce tea to the diet of children <2 years of age [68,69,70,71,72] and thus caffeine guidelines targeting this age group are highly relevant. Only thirteen country FBDG, spanning Asia and the Pacific, Europe, Latin America, and North America, advise pregnant/nursing women to avoid CCB. Eight of these advised specific caffeine limits, which ranged from 200 to 300 mg/day. Small epidemiological studies report that over 60% of women drink caffeine-containing beverages during pregnancy, but total amounts are generally below advised limits [23,73,74,75,76,77]. For adults, Denmark, U.K., Portugal, Canada, and USA advise to limit caffeine intake to 300 or 400 mg/day. FBDG for Australia, Indonesia, New Zealand, Denmark, Hungary, Malta, Colombia, USA, and Canada state specific concerns for energy drinks, generally defined as any drink with >150 mg of caffeine/liter, but often contain other bioactive ingredients and sugar [16,78]. Some guidelines to avoid or limit caffeine intake were based on human or animal studies of pregnancy outcomes, fetal development, and acute caffeine effects (including diuresis, see below). Other guidelines were in place as a safety-precaution since the long-term adverse effects of caffeine are not clear.

Water is essential for life and thus a staple recommendation in all FBDG. Coffee, tea, and yerba mate are naturally non-caloric beverages and currently make important contributions to total fluid intake for many countries [79,80,81,82], but whether they are suitable substitutes for plain water varies by country guidelines and likely reflects the nutritional priorities of the country. For example, FBDG of African countries stress the importance of consuming “enough safe” water as opposed to listing adequate water-substitutes. They were nevertheless concerned about the iron absorption inhibitory effects of tannins present in coffee and tea [83,84,85,86,87], as were the FBDG of several countries of Asia and the Pacific. For some FBDG, whether coffee or tea were adequate water-substitutes was often dependent on whether the diuretic effects of caffeine were considered significant by the country. European and North American countries rarely noted these diuretic effects.

Caffeine-containing soda is a major contributor to sugar intake along with other SSB and implicated in obesity and other metabolic disease around the globe [88]. Guidelines concerning reductions in soda are thus geared towards reducing sugar intake as opposed to monitoring caffeine intake. Coffee and tea become significant sources of added sugar and energy in the diet for countries such as China, Korea, Malaysia, Spain, Italy, Brazil, and Uruguay that prefer to prepare coffee and tea with sugar and cream, or for countries where instant coffee mixes are highly consumed [37,43,44,82,89,90,91,92,93]. These habits are often overlooked and may off-set any benefits that coffee and tea might offer over other beverage types [89,92]. In our review of guidelines, Sri Lanka, Thailand, Qatar, Bulgaria, France, Greece, Italy, Malta, Poland, Turkey, Brazil, Mexico, and the USA advised careful attention to the amount of sugar added to coffee and tea.

While there is currently no evidence of health benefits for caffeine-added beverages, recent reviews concerning coffee, and perhaps tea, suggest some benefits with coffee and tea consumption [6,10]. Some FBDGs make reference to these benefits and a few of these also provide specific guidelines. In 2015, the USA dietary guidelines committee reviewed the literature concerning coffee, specifically, as well as total caffeine on health. Potential benefits of three to five cups of coffee/day were discussed in the committee’s Scientific Report [25]. The favorable message, however, could not yet be applied to children or pregnant women or for an equivalent amount of total caffeine (from any source). The Netherlands also point to benefits of green and black tea consumption and *recommend* three cups/day. Interestingly, Poland *discourages* consumption of black tea in particular, and Latvia *discourages* the use of coffee or tea for hydration, in part, for mental health and heart disease prevention. India also provides an in-depth look at coffee, tea, and caffeine. Coffee and caffeine are viewed negatively and potential benefits with tea are off-set by its caffeine content. Mexican guidelines classify beverages from the most (level 1) to the least (level 5) healthy according to their energy content, nutritional value, and risks to health. Coffee and tea (without sugar) are level 3 beverages, *limited* to four cups/day. No African studies encourage coffee and tea consumption for health. Taken together, inconsistencies concerning health benefits (and risks) of coffee and tea consumption were observed across FBDG and this may be due to the breadth of research on the topic (function of FBDG development date and country-relevancy) or nutritional priorities of the country.

Most guidelines pertaining to dietary caffeine are evidence-based but there are some exceptions. Portugal’s guidelines, for example, state “in tea, the absorption of caffeine is slower than in coffee, which means the stimulating effect is lower but lasts longer.” Albania guidelines advise menopausal women to avoid coffee (among other foods/beverages) because it worsens “warming.” Peer-reviewed literature supporting these statements were often not available. Missing from guidelines was information on known between-person variation in caffeine metabolism, resulting from lifestyle or genetic factors [94,95]. However, despite enthusiasm for “personalized-caffeine recommendations,” further studies are warranted before they can be included in FBDG.

While FBDG help individuals optimize their caffeine habits, many countries regulate caffeine intake at the food manufacturing level by setting limits to the amount of caffeine added to foods [78,96]. Several countries have specifically enacted measures to regulate the labeling, distribution, and sale of energy drinks [2,8,97,98]. For example, Denmark, Turkey, Norway, Uruguay, Sweden, Lithuania, Latvia, and Iceland have banned or restricted sales to children or those <18 years of age, while Hungary and Mexico apply an additional tax to energy drinks [20,21,99,100]. The USA, Canada, and Mexico have further restrictions on the sale of caffeinated alcohol beverages [101,102]. In view of the health risks associated with the widespread consumption of SSB, many national governments have also taken action to reduce consumption of SSB [20,21,103,104]. While these actions are not targeting caffeine, per se, they are targeting a subset of SSB that contain caffeine which include colas and energy drinks. Unfortunately, all policies in place to regulate caffeine intake are challenged by the fact that major dietary sources of caffeine (i.e., coffee and tea) are exempt since they naturally contain caffeine [105,106].

Our data collection strategy for dietary caffeine guidelines was systematic and comprehensive but may be incomplete. We relied on FAO as a starting point which may have missed FBDG of certain countries or may not have been updated with the latest FBDG. Our approach offered several opportunities to address the latter. Our efforts to search and contact secondary resources was often met with limited success. As described elsewhere, the Euromonitor Passport is not a scholarly database and the data have similar limitations to official government trade and economic statistics [107]. Euromonitor data capture sales volume only, an imperfect measure of consumption because it does not capture products distributed through informal food systems or wastage [108]. Moreover, some beverage categories are not exclusively CCB. For example, sports drinks without caffeine are consumed in greater quantities than energy drinks and thus contributions to overall CCB by the “sports drinks and energy drinks” is likely overestimated. However, these data are abundant, less biased than survey data, and they have been consistently reported across countries and time using standardized measures [107]. Despite these limitations, the current review is a starting resource for country-level guidelines and consumption data pertaining to dietary caffeine.

In summary, FBDG provide an unfavorable view of caffeinated-beverages by noting their potential adverse/unknown effects on special populations as well as their diuretic, psycho-stimulating and nutrient inhibitory properties. Few FBDG balanced these messages with recent data supporting potential benefits of specific beverage-types. FBDG can serve to guide a wide range of food and nutrition policies and programs with the unique opportunity to favorably impact diets and the food system [17]. FBDG undergo review and revisions in keeping with changes in nutrition priorities of a country and advancements in nutrition research. We therefore anticipate modifications to guidelines pertaining to caffeine in future releases of FBDG.

## Figures and Tables

**Table 1:** Country Specific Guidelines for Dietary Caffeine.

Country Publication Date	*FAO key Messages* and Published Guidelines [Other Resources and Personal Communication]
**AFRICA**	
Benin ^LI^ 2015	*Drink carbonated drinks and sugar-sweetened beverages in moderation. These kinds of drinks only provide sugar and can promote obesity and diabetes*.
Nigeria ^LMI^ 2001	Adults, elderly, pregnant, breast-feeding: Avoid coffee and tea during meals since these beverages hinder iron absorption. Diabetics: avoid coffee if hypertensive.
Seychelles ^HI^ 2006	*Consume sugar, sugary foods, and sugary drinks in minimal amounts*.
Sierra Leone ^LI^ 2016	*Use sugars and foods and drinks made with sugar in moderation*. Limit intake of beverages such as coffee and alcohol. Taken in excess, these will contribute to too much phosphorous, which depletes calcium levels.
South Africa ^UMI^ 2012	*Use sugar and foods and drinks high in sugar sparingly*. Avoid giving tea, coffee, and sugary drinks and high-sugar, high-fat salty snacks to baby/child (6–36 months). Low nutrient-dense liquid and energy-dense sugar-sweetened, high-fat, and salty snacks exacerbate poor nutrient intake and displace nutrient-dense food in the diet. Tea and coffee have low nutrient content and polyphenols that inhibit iron bioavailability. Drink lots of clean, safe water including drinking water and other foods/beverages that contain water. Biological availability of water depends on the presence of various ingredients in foods and beverages (i.e., salt and carbohydrates accelerate water absorption; caffeine and alcohol have diuretic effects). Research has shown that the consumption of caffeinated beverages, such as tea and coffee, can add to the daily water balance in individuals who are used to ingesting these beverages. Acute increases in urine output only occur in individuals who are not accustomed to regular consumption of caffeinated beverages.
**ASIA and the PACIFIC**	
Afghanistan ^LI^ 2015	*Reduce sugar intake and avoid sweet carbonated beverages*.
Australia ^HI^ 2013	Drink plenty of water: Tea and coffee provide water, although they are not suitable for young children and large quantities can have unwanted stimulant effects in some people. Consumption of drinks with added sugars, such as soft drinks and cordials, fruit drinks, vitamin waters, and energy and sports drinks can increase the risk of excessive weight gain in both children and adults. Limit intake of foods and drinks containing added sugars such as confectionary, sugar-sweetened soft drinks and cordials, fruit drinks, vitamin waters, and energy and sports drinks.
Bangladesh ^LMI^ 2013	*Consume less sugar, sweets, or sweetened drinks*. Drink coconut water and fresh fruit juices instead of carbonated drinks. Pregnancy and lactation: Iron supplements are poorly absorbed when they are taken with beverages such as coffee or tea or simultaneously with calcium supplements. They should be taken after a meal, preferably after breakfast or after lunch.
China ^UMI^ 2016	For lactating women, overconsumption of coffee should be avoided. Consumption of sugar-sweetened beverages should be limited in all age groups.
Fiji ^UMI^ 2013	Drinks like tea and coffee are diuretics, which make you produce more urine than usual. Therefore, if you drink lots of tea and coffee, you still need to drink plenty of water. Fizzy and sugary drinks are also not a good way to get your fluid. The high sugar content can cause unwanted weight gain or take the place of more nutritious foods in your diet.
India ^LMI^ 2011	Lactating women: Excess intake of beverages containing caffeine like coffee and tea adversely affect fetal growth, and hence, should be avoided. Since drugs (antibiotics, caffeine, hormones, and alcohol) are secreted into the breast-milk and could prove harmful to the breast-fed infant, caution should be exercised by the lactating mother while taking medicines. Drink plenty of water and take beverages in moderation: Tea and coffee are popular beverages. They are known to relieve mental and muscular fatigue. This characteristic stimulating effect is due to their caffeine content. Caffeine stimulates the central nervous system and induces physiological dependence. Generally, low doses (20–200 mg) of caffeine produce mild positive effects like a feeling of well-being, alertness, and being energetic. Higher doses (>200 mg) can produce negative effects like nervousness and anxiety, especially in people who do not usually consume caffeine-containing beverages. Therefore, moderation in tea and coffee consumption is advised so that caffeine intake does not exceed the tolerable limits. Tannin is also present in tea and coffee and is known to interfere with iron absorption. Hence, tea and coffee should be avoided at least for one hour before and after meals. Excess consumption of coffee is known to increase blood pressure and cause abnormalities in heartbeat. In addition, an association between coffee consumption and elevated levels of total and low-density lipoprotein (LDL) cholesterol (“bad” cholesterol), triglycerides, and heart disease has been demonstrated. Therefore, individuals with heart disease need to restrict coffee consumption. Also, those who experience adverse effects from caffeine should stop drinking coffee. Besides caffeine, tea contains theobromine and theophylline. These are known to relax coronary arteries and thereby promote circulation. Tea also contains flavonoids and other antioxidant polyphenols, which are known to reduce the risk for coronary heart disease and stomach cancer. However, as a result of its caffeine content, excess tea consumption is deleterious to health. Decaffeinated coffee and tea are being marketed to obviate the adverse effects of caffeine.
Indonesia ^LMI^ 2014	One should limit consumption of overripe fruits and sugary juice drinks to control blood glucose. It should be noted that other food containing simple carbohydrate (flour, bread, soy sauce), sweet fruits, juice, soda drinks, etc., also contain sugar. Caffeine, if consumed by pregnant women, has a diuretic and stimulant effect. Thus, pregnant women may experience increased urination which may lead to dehydration, and increase in blood pressure and heart rate if their coffee intake is not controlled. Other sources of caffeine are chocolate, tea, and energy supplement drinks. In addition to caffeine, coffee also contains a substance that may inhibit iron absorption. Caffeine consumption in pregnant women may affect fetus growth and development, since the metabolism is not fully developed. The National Institute of Health USA (1993) recommends safe caffeine consumption for pregnant women as 150–250 mg/day or two cups of coffee/day. Thus, it is recommended for pregnant women “to be wise in consuming coffee, limit your intake within safe range, i.e., 2 cups of coffee/day or avoid at all, as there is no nutrition content in the coffee.” Children should not regularly drink sweet or soda drinks as they have high sugar contents. For daily liquid consumption, it is recommended for children to drink 1200–1500 mL water/day. Breastmilk may contain caffeine from the coffee consumed by nursing women. This may have unfavorable effects on babies since their metabolism are not fully developed to digest caffeine. Caffeine consumption in nursing women may be related with low production of breastmilk. Based on research in Harvard University, safe consumption of caffeine in nursing women is 300 mg/day or three cups of coffee/day. Based on research in Mayo Clinic Rochester Minnesota USA, consumption exceeding 300 mg/day will lower iron content in breastmilk by 30% compared to nursing women who do not take caffeine.
Japan ^HI^ 2010	*Drink plenty of water and tea. Moderate consumption of highly processed snacks, confectionary, and sugar-sweetened beverages*.
Malaysia ^UMI^ 2010	Consume foods and beverages low in sugar. Caffeine is naturally present in coffee, tea, and chocolate, and added to colas and other beverages. It has long been thought that consumption of caffeinated beverages, because of the diuretic effect of caffeine on reabsorption of water in the kidney, can lead to loss of body water. However, available data are inconsistent. Caffeine-containing beverages did not increase 24 h urine volume in healthy, free-living men when compared with other types of beverages for instance water, energy-containing beverages, or theobromine-containing beverages. In aggregate, available data suggest that higher doses of caffeine (above 180 mg per day) have been shown to increase urinary output, perhaps transiently, and that this diuretic effect occurs within a short time period. Hence, unless additional evidence becomes available indicating cumulative total water deficits in individuals with habitual intakes of significant amounts of caffeine, caffeinated beverages appear to contribute to the daily total water intake similar to that contributed by non-caffeinated beverages (Food and Nutrition Board, 2004).
Nepal ^LI^ 2012	*Consume less sugar, sweets, and sweetened drinks*.
New Zealand ^HI^ 2008–2013	0–2 years Coffee, tea, other caffeine-containing drinks, smart or energy drinks, herbal teas, and alcohol are unsuitable for infants and toddlers. Caffeine is a central nervous system stimulant, which can cause irritability and restlessness. Tea is not recommended because its tannin content inhibits the absorption of iron from the gut. This impact was confirmed by a New Zealand study that observed a significant association between tea drinking in infants and the presence of an iron deficiency. Herbal teas can have adverse effects on infants and toddlers, so are not recommended. 2–18 years Children and young people should limit their intake of foods and drinks containing caffeine. Caffeine is a psychoactive stimulant drug that acts on the central nervous system, alters brain function, acts as a diuretic, and elevates blood pressure and metabolic rate. Acute adverse effects from caffeine that have been identified include anxiety, headaches, insomnia, irritation of the gastrointestinal tract, nausea, and depression. Long-term adverse effects from caffeine are not clear. Children may be more sensitive to adverse effects of caffeine than other groups in the population. An upper exposure of 2.5 mg/kg of body weight per day has been suggested as a cautious toxicological limit on which to base risk assessment for children, on the grounds of limited evidence. Energy drinks and energy shots contain large amounts of caffeine, a psychoactive stimulant drug, and often large amounts of sugar. They are not recommended for children and young people. Coffee and tea are not recommended for children less than 13 years of age. If drinking tea and coffee, it is recommended young people (13–18 years) limit their intake to one to two cups per day. They should avoid drinking tea at meal times, as this drink contains tannins and polyphenols, which can inhibit the absorption of nutrients, such as iron. Pregnant and breast feeding Caffeine is a mild central nervous system stimulant, present in chocolate and beverages such as coffee, tea, energy drinks, and cola. Caffeine readily crosses the placenta to the fetus and has also been found to stimulate metabolic rate. Many over-the-counter medications, such as cold and allergy tablets, headache medications, diuretics, and stimulants, also contain some caffeine. High doses of caffeine in pregnancy have been associated with increased risk of congenital abnormalities, pregnancy loss, low birth weight, and behavioral problems. Decaffeinated coffee appears to have no effect on birth weight. The effects of caffeine may be synergistic with those of smoking and alcohol. Caffeine is transferred into breast milk. The infant metabolizes and excretes caffeine slowly. High caffeine load in breast milk may lead to irritability and poor sleeping patterns and, occasionally, increased bowel activity. The benefits of breastfeeding outweigh any risks associated with the presence of caffeine in breast milk, however. Consuming caffeine-containing beverages immediately after the baby has fed will limit the amount of caffeine in the next feed. The UK Food Standards Agency advises pregnant women to limit their intake of caffeine to 300 mg per day. Breastfeeding women should consider their caffeine intake if the infant is irritable or wakeful. Older people Although there is some evidence that a high caffeine intake is a risk factor for fracture frequency or bone loss, there is also evidence to the contrary. High intakes of caffeine do increase urinary calcium excretion. Several large cohort studies have reported small but significant increases in either fracture frequency or bone loss associated with increased caffeine intake. Other studies have found no such association. Moderate caffeine intake is not associated with increased bone loss, and so a prudent recommendation would be for adequate dietary calcium intake together with moderate caffeine consumption in older adults. A “moderate” level of caffeine intake seems likely to be 300 mg or less of caffeine, which is equivalent to approximately: one large long black, three cappuccinos, four cups of plunger coffee, or six cups of tea. Adults Make plain water your first choice over other drinks; black tea and coffee are also popular and there is some evidence that both can provide benefits for health such as antioxidative properties. Tea and coffee both contain caffeine (a stimulant) and tea contains tannins, which lower the amount of iron that the gut absorbs. Therefore, the Ministry of Health recommends drinking only moderate amounts of tea and coffee.
Republic of Korea ^HI^ 2010	Pregnant mothers should minimize coffee, black tea, soda, and chocolate. Young children should minimize the consumption of crackers/cookies, carbonated beverages, and fast food. Young adolescents should minimize instant and fast foods and carbonated beverages and should avoid junk food.
Sri Lanka ^LMI^ 2011	*Consume less sugar, sweets, or sweetened drinks*. Tea without milk and sugar will have certain advantages as they have some antioxidants that will help to improve health. However, it is advisable to avoid tea or coffee closer to a main meal, as it will reduce iron absorption.
Thailand ^UMI^ 2008–2010	Most food eaten on a daily basis as a main dish or dessert contain sugar. We also take additional sugar from soft drinks, candy, toffee, jelly, syrup, and sugar added to tea, coffee, and other beverages. Therefore, we often add excessive amounts of energy to our regular diet. Children who eat sugary foods often have a lower appetite and are prone to tooth decay. Thus, sugary foods should be limited in their diets.
Vietnam ^LMI^ 2013	*Increase physical activity, maintain an appropriate weight, abstain from smoking, and limit your consumption of alcoholic/soft drinks and sweets*.
**NEAR EAST**	
Iran ^UMI^ 2015	*Reduce your consumption of sugar, sweet foods and beverages, and soft drinks*. *During the day drink water and unsweetened beverages frequently*.
Lebanon ^UMI^ 2013	*Limit intake of sugar, especially added sugar from sweetened foods and beverages*. Avoid drinking tea, coffee, and caffeine-containing carbonated beverages with meals, as food components in these beverages may decrease the absorption of iron in food. The consumption of the below food components can increase the urinary loss of calcium Salt (in excessive amounts) Caffeine (in amounts coming from more than three to four cups of coffee per day). Water is the preferred fluid to fulfill the body’s daily fluid needs and is followed (in decreasing order) by: tea and coffee, low-fat and fat-free milk, non-sugar sweetened beverages that contain some nutritional benefits (such as fruit and vegetable juices), sugar-sweetened and nutrient-poor beverages (such as sweetened fruit juices and soft drinks). Although tea and coffee contain a good amount of water, these fluids contain caffeine, which increases urine excretion. As such, these fluids lead to a loss of water from the body if consumed in high amounts. Consumption of sugar-sweetened beverages has been linked to overweight, obesity, type 2 diabetes, and dental caries. Since fluid-derived energy is an important consideration in weight gain, drinking water should be the preferred beverage for hydration purposes.
Qatar ^HI^ 2015	Limit sweetened food and beverages. Be aware of the amounts of sugar in hot and cold coffee beverages served in cafes. Avoid sweetened drinks such as soda, energy drinks, fruit drinks, vitamin waters, and sports drinks. Allow 30 min after meals before drinking tea to allow for absorption of iron from foods.
**EUROPE**	
Albania ^UMI^ 2008	Infants in their first year of life Iron absorption is decreased with drinking tea and coffee Adults Consume 1–2 liters of beverages per day choosing those with no sugar added but potable water, mineral water, and fruit or plant teas. Take limited quantities of beverages containing caffeine (coffee, black or green tea, etc.). Women breastfeeding Avoid alcoholic beverages and products which contain caffeine. Women in menopause It is important to stay away from the alcoholic beverages, coffee, and spicy food because those influence in worsening the warmth. Children 2–3 years old Consume at least three glasses of sugarless juices (water, mineral water, original plant tea, fresh watered juicy fruits, no sugar added, etc.) per day. The beverages should not contain caffeine or alcohol. Children 4–6 years old Consume at least three to four glasses of sugarless juices per day. The beverages should not contain caffeine like teas or cola. Teenagers 13–18 years old Consume at least five to six glasses of sugarless juices per day. The beverages should not contain caffeine like teas or cola and alcohol.
Austria ^HI^ 2010	*Non-alcoholic beverages: Drink at least 1.5 liters of fluid, preferably low-energy drinks in the form of water, mineral water, unsweetened fruit or herbal teas, or diluted fruit and vegetable juices. A daily moderate consumption of coffee, black tea (three to four cups) and other caffeinated beverages is acceptable*. *Processed foods high in fat, sugar, and salt: Some processed foods (such as sweets, pastries, fast food products, snacks, and soft drinks) are high in fat, sugar, and salt and are less desirable nutritionally. They should be consumed sparingly—a maximum of one small serving a day*. There is no objection about the daily moderate consumption of coffee, black tea (three to four cups) and other caffeinated drinks. Is coffee a liquid robber? In “normal” quantities (up to four cups/day) it is not.
Belgium ^HI^ 2005	*Drink at least 1.5 liters of water every day (water, coffee, tea, etc.)*. Pregnant women Avoid alcohol, tobacco, and large amounts of tea and coffee. 3 to 12 years Water is preferred over other beverages since the latter often contain a lot of sugar. 13 to 18 years Water, tea, or coffee are components of a “good breakfast.” 60 and older Water, (weak) coffee, and tea are encouraged at each meal and throughout the day to maintain hydration. “Food for brain”: tea and coffee are sources of antioxidants.
Bulgaria ^UMI^ 2006	*Limit the consumption of sugar, sweets, and confectionery, and avoid sugar-containing soft drinks*. The high intake of sugar and sugar-containing foods and beverages leads to being overweight and obesity, which themselves increase the risk for hypertension, cardiovascular diseases, and type 2 diabetes. Try to drink tea or coffee without sugar. Prefer honey or brown sugar for sweetening.
Croatia ^HI^ 2002	Black coffee can reduce calcium absorption.
Cyprus ^HI^ 2007	*Reduce the consumption of sugar, preferring appropriate beverages and foods with reduced or no sugar*.
Denmark ^HI^ 2013	Maximum amount of caffeine for an adult/day is 400 mg. Caffeine for children and youngsters is discouraged. Pregnant and breastfeeding women are encouraged to minimize their intake of caffeine. Furthermore, they are discouraged from drinking any energy drinks.
Estonia ^HI^ 2006	*Limit the consumption of sweets and soft drinks*.
Finland ^HI^ 2014	*Decrease consumption of soft drinks and sweet juices*. You should avoid having sugary drinks regularly, as they are associated with obesity and the risk of developing type 2 diabetes. A high intake of sugary drinks also affects your dental health.
France ^HI^ 2011	*Limit the consumption of sugar and foods high in sugar (soft drinks, candies, chocolate, pastries, desserts, etc.)* Beverages: To quench your thirst, water is the only essential drink. You should drink at least a liter and a half a day, during and between meals, as it is or in the form of hot drinks (tea, herbal tea, coffee). If you are fond of soft drinks or sweet drinks, try to content yourself with one drink a day or only two or three at a party. The sugar contained in these drinks does not decrease your appetite and makes you easily gain weight. Consume sugary drinks with moderation. Instead, choose fruit juices without added sugar. If you drink a lot of soft drinks, and do not manage to replace with water, drink light versions to reduce your sugar intake. In tea or herbal teas, a little spoon of honey is always better than a cube of sugar. For children, it is best to regularly offer tap water, spring water, or mineral water (flat or carbonated), with plant extracts and sugar-free; plain or flavored milk with cocoa, vanilla, orange blossom, or syrup (fruit based concentrates); or pure fruit juice (no added sugar) or a squeezed fruit juice.
Georgia ^UMI^ 2005	*Do not drink tea while eating plant meals rich in iron (e.g., vegetables, legumes), because tea limits the availability of non-heme iron*. *Sweet drinks can be replaced by sufficient amounts of unsweetened liquids, e.g., boiled water*.
Germany ^HI^ 2013	Drink water with or without carbon dioxide and low-energy drinks. Drink sweetened drinks rarely. These are energetic and can increase the emergence of obesity.
Greece ^HI^ 1999	Always prefer water over soft drinks. Non-alcoholic beverages, including sodas, have not been conclusively linked to health effects. Fruit juices are likely to share some of the benefits of fruits, whereas other beverages have been criticized for their high content in simple carbohydrates. Simple sugars are plentiful in deserts, and also exist, or are added, in beverages, like coffee, tea, fruit juices, soft drinks, and colas. Simple sugars have glycemic effects, mainly comparable to or less than those of starch from cooked foods.
Hungary ^HI^ 2004–2016	*Avoid the frequent consumption of foods or drinks rich in added sugar. To quench your thirst, drink water or mineral water instead of sugary drinks*. Children: Consumption of energy drinks is not recommended because it has high caffeine and high sugar content.
Ireland ^HI^ 2012	*Limit high fat, sugar, and salt foods from the top shelf of the pyramid to no more than once or twice a week (includes soft drinks and juice)*. Fluids: Water is the best drink for hydration and the safest for teeth. At least 8–10 cups of fluid are needed every day, and this can come from fluids in the foods eaten as well as water, milk, tea, and coffee. Other sources of excessive sugar in the form of sweets, soft drinks, cakes, biscuits, and confectionery are not integrated with nutritious high-fiber foods. Limiting these foods will help to lower calories, fat, sugar, and salt. Although “diet” soft drinks (sugar free) can be used sometimes for variety, they are acidic and if taken too frequently, they can still harm teeth. Pregnant women: Caffeine in the mother’s diet can reach the baby and may be harmful. High caffeine intakes during pregnancy are not advisable, and mothers should aim to keep their caffeine intake below 200 mg of caffeine per day (an accompanying table provides caffeine content of the most common caffeine-containing foods and drinks). Also limit consumption of caffeine while breastfeeding.
Israel ^HI^ 2008	Eat/drink sweets, snacks, and sweetened drinks sparingly.
Italy ^HI^ 2003	*Consume appropriate amounts of sugars, sweets, and sugar-sweetened beverages*. The balance of water must be maintained by drinking essentially water, both tap and bottled, safe and controlled. Remember that certain drinks (such as orange juice, sodas, fruit juices, coffee, and tea), in addition to providing water, also bring other substances that contain calories (for example, simple sugars) or that are pharmacologically active (for example caffeine). These should be used in moderation.
Latvia ^HI^ 2003–2008	*Limit consumption of salt and sugar and products containing them*. Do not wait until you feel thirsty—use liquid regularly! The day should consist of eight glasses of liquid including water and herbal teas. [The Centre for Disease Prevention and Control infographics stresses the importance of using non-sweetened and non-caffeinated drinks like pure water (2 liters a day) or herbal tea for hydration needs. Usage of these liquids instead of coffee or tea is also recommended for mental health and heart disease prevention. It is recommended for pregnant women to drink water every day and limit caffeine consumption to 200 mg per day. Exclusion of energy drinks during this period is also recommended. As coffee and tea reduces iron absorption, reduced intake of these drinks is recommended whilst using iron supplements. Limited coffee and tea usage for vegetarians is also recommended, especially during meals as these drinks reduce calcium absorption]
Malta ^HI^ 2016	*Avoid adding sugar to your tea or coffee. Avoid energy drinks. Avoid soft and sweetened drinks especially in children*. Avoid energy drinks: Energy drinks generally have a high sugar and caffeine content as well as other stimulant substances. They can easily contribute to an excess energy intake and may cause a variety of problems such as headaches, restlessness, insomnia, stomach upset, fast heartbeat, tooth decay, and anxiety.
Netherlands ^HI^ 2016	*Drink three cups of tea daily*. *Replace unfiltered coffee by filtered coffee*. *Minimize consumption of sugar-containing beverages*. Tea: In the context of this advisory report, the term “tea” covers green tea and black tea. Green tea comes from the tea plant, but unlike black tea, it has not undergone oxidation. Herbal teas and, for example, rooibos are outside the scope of this report. The Committee concludes that it has been convincingly demonstrated that the consumption of tea reduces the risk of stroke. That conclusion is based on the fact that randomized controlled trials (RCTs) show that three cups of green tea or five cups of black tea a day reduce blood pressure, while the consumption of tea is associated with a lower risk of stroke in cohort studies. In addition, the consumption of black tea and the consumption of green tea are plausibly associated with a lower risk of diabetes. Coffee: The way that coffee is prepared, whether it is filtered or not, makes a difference to its influence on health. That is because filtering can remove the cholesterol-raising substances cafestol and kahweol from coffee. In the context of this advisory report, “filtered coffee” covers coffee made using a filter machine, coffee made using coffee pods, instant coffee, and vending-machine coffee made using liquid coffee concentrate. Unfiltered coffee includes boiled coffee, cafetière coffee, Greek coffee, and Turkish coffee. Espresso and coffee from vending machines that use fresh coffee may count either as filtered or as unfiltered, depending on the type of machine, the type and amount of coffee, and the type of filter used. The Committee concludes that it has been convincingly demonstrated in RCTs that unfiltered coffee increases LDL cholesterol, which is known to be a causal risk factor for coronary heart disease. Coffee consumption is associated with lower risks of coronary heart disease, stroke, and diabetes in cohort studies, which relate predominantly to filtered coffee consumption. Sugar-containing beverages: Examples include fruit juice drinks and “nectars,” carbonated drinks (“pops” and “sodas”), ice tea, vitamin-fortified water, and sports drinks made by the addition of sugar. The Committee concludes that it has been convincingly demonstrated that the consumption of drinks with added sugar increases the risk of diabetes. That conclusion is based on the fact that RCTs have shown that drinks with added sugar increase body weight, while cohort studies indicate an association of the consumption of drinks with added sugar with a higher risk of diabetes.
Norway ^HI^ 2014	*Find the right balance between how much energy you consume through food and drink and how much energy you use by being physically active*. *Limit your consumption of food and drink with a high sugar content*. People at risk of developing heart disease and diabetes should limit their consumption of non-filtered coffee. It is recommended that drinks with added sugar or caffeine not be offered in schools.
Poland ^HI^ 2010	*Moderate your intake of sugar and sweets*. Drink tea and coffee in moderation. It is better to choose green tea, fruit tea, or herbal tea and rarely drink black teas and coffee. Exclude or limit sweetened beverages, especially sodas (carbonated drinks). Their excess in diet favors even more occurrence of overweight and obesity than consumption of great amounts of sugar and sweets. Carbon dioxide can suppress thirst. Tea is a good source of water, which you can drink but in smaller amounts. Fruit and herbal teas are best, rarely drink dark tea, which should not be too strong. Try not to sweeten tea, and if you do not like the bitter taste, decrease the amount of added sugar.
Portugal ^HI^ 2003	*Prefer water to beverages containing added sugar, alcohol, and caffeine*. The recommended minimum consumption of liquids per day is 1.5 to 3 liters, depending on the activity and state of health of the individual. Although water is the best drink to satisfy your thirst, you can also drink other drinks that do not contain added sugar, alcohol, or caffeine. Natural fruit juices and caffeine-free teas are examples of these beverages. Coffee and some teas and soft drinks contain caffeine, a stimulant substance whose intake should be limited to a maximum of 300 mg per day. In the case of children adolescents and their consumption is discouraged. Despite the designation, decaffeinated beverages are not completely exempt of this substance. In tea, the absorption of caffeine is slower than in coffee, which means the stimulating effect is lower but lasts longer.
Romania ^UMI^ 2006	*Eat highly processed foods high in sugar sparingly*. Caffeine, nicotine, and warm foods also increase the thermic effect of food. Various seeds, unprocessed grains, sprouted wheat and wheat bran, nuts, vegetables, green vegetables, hard water, coffee, tea, and cocoa are good sources of magnesium. Pregnant women Iron supplements must be administered between meals, preferable with liquids containing ascorbic acid (increases the iron absorption), avoiding concurrent administration of tea, milk, or coffee. Caffeine: There are controversies about an acceptable level of the caffeine intake during pregnancy. In USA, the Food and Drug Administration (FDA) recommends limiting the intake of caffeine during pregnancy and if possible complete avoidance, given the teratogenic effects seen in animal studies. In humans, large doses of caffeine (over 300 mg/day) are associated with low birth weight. Caffeinated beverages should also be avoided while breastfeeding. Elderly Avoid alcohol abuse, excess intake of caffeine and unnecessary medications. Tea and coffee have no contribution to calorie intake unless milk, cream, or sugar is added. They contain caffeine and theobromine and their excess use may cause insomnia and irritability. Tea is an important source of bioflavonoids with antioxidant properties that might protect against cardiovascular disease. Cocoa contains significant amounts of iron, proteins, fats, and carbohydrates; however, because of the quantities in which it is usually consumed, it loses its nutritional value.
Slovenia ^HI^ 2011	*Consume enough fluids, preferably drinking water, mineral water, unsweetened fruit or herbal teas or diluted fruit and vegetable juices*.
Spain ^HI^ 2008	Avoid excessive consumption of sugary soft drinks and juices with added sugar. The studies warn about the relationship between the excessive consumption of these soft drinks and the increase in childhood obesity. Do not consume them as a substitute for water.
Sweden ^HI^ 2015	*Less sugar - Hold back on the sweets, pastries, ice creams, and other products containing lots of sugar. Cut back on sweet drinks in particular*. Water is by far the best drink for quenching thirst—much better than fizzy drinks, juice, soft drinks, and sports drinks. Sweet drinks in particular increase the risk of obesity as they contain lots of calories but do not make you feel full.
Switzerland ^HI^ 2011	Beverages: Consume 1–2 liters per day, preferably in the form of unsweetened drinks such as tap water/mineral water or fruit/herb tea. Caffeinated drinks, such as coffee, black tea, and green tea, can also count towards your fluid intake.
Turkey ^UMI^ 2006	Reduce the consumption of sugary beverages and sweets and choose foods containing less sugar. Instead of sugar-added soft drinks, please prefer skim milk, ayran (watered yogurt), and kefir. Instead of drinking beverages containing sugar, water should be preferred. Drink sugar-free tea and herbal teas.
United Kingdom ^HI^ 2016	*If consuming foods and drinks high in fat, salt, or sugar, have these less often and in small amounts*. Aim to drink six to eight glasses of fluid every day. Water, lower fat milk, and sugar-free drinks including tea and coffee all count. Fruit juice and smoothies also count towards your fluid consumption, although they are a source of free sugars and so you should limit consumption to no more than a combined total of 150 mL per day. Swap sugary soft drinks for diet, sugar-free, or no added sugar varieties to reduce your sugar intake in a simple step. The European Food Safety Authority (EFSA) opinion confirms the safety of daily caffeine intakes of up to 3 mg per kg of body weight for children and adolescents (3–18 years) and up to 400 mg for adults.
**LATIN AMERICA and the CARIBBEAN**	
Antigua and Barbuda ^HI^ 2013	*Reduce the intake of food and drinks that are high in sugars and fats*.
Argentina ^UMI^ 2015	*Limit the consumption of sugary drinks and foods high in fats, sugar, and salt*.
Bahamas ^HI^ 2002	Eating too much high-seasoned and sweet foods increases your risk of developing heart disease, high blood pressure, and type II diabetes. Sodas, fruit-flavored drinks, and desserts like guava duff and tarts contain lots of sugar.
Barbados ^HI^ 2017	*Choose food and beverages with less added sugar every day*. Many foods and beverages with added sugar are high in calories and have little nutritional value. These extra calories can contribute to overweight and obesity. High sugar intake can also increase blood pressure and risk of death from cardiovascular disease.
Belize ^HI^ 2012	Replace sweet drinks with water. Add less sugar when preparing foods and drinks. Foods that contain a high amount of sugar include soft drinks, box drinks, cakes, ice cream, puddings, sweets, jams and jellies, and condensed milk. Benefits: better weight control, better control of blood sugar levels, and fewer problems with dental caries/tooth decay.
Bolivia ^LMI^ 2013	*Avoid the over consumption of sugar, sweets, sodas, and alcoholic drinks*. *Reduce the consumption of tea and coffee, replacing them with milk, fruit juices or “apis”*. Avoid the exaggerated consumption of sugar, sweets, soft drinks: Exaggerated consumption of these products cause tooth decay and deteriorate health. Certain substances such as caffeine and alcohol have a diuretic effect and increase the urinary losses of water and electrolytes. Adequate water intake can be covered not only with water, but also with food or liquids that do not contain caffeine and alcohol.
Brazil ^UMI^ 2014	*Avoid consumption of ultra-processed foods*. *Because of their ingredients, ultra-processed foods, such as salty fatty packaged snacks, soft drinks, sweetened breakfast cereals, and instant noodles, are nutritionally unbalanced. As a result of their formulation and presentation, they tend to be consumed in excess, and displace natural or minimally processed foods. Their means of production, distribution, marketing, and consumption damage culture, social life, and the environment*. Examples of natural/minimally processed foods: tea, herbal infusions, coffee, and tap, spring, and mineral water. Breakfast examples: Fruits and coffee with milk are a constant part of the first meal of the day. Water: Pure water (or, as preferred by some people, “seasoned” with lime slices or mint leaves) is the best option. Brazilians also consume water in the form of coffee and tea, in which case sugar should be reduced to a minimum or not added at all.
Chile ^HI^ 2013	*If you want to maintain a healthy weight, avoid eating sugar, sweets, sugar-sweetened juices, and beverages*. The [lactating] mother should be well fed, eat healthy, drink water, and not consume any alcoholic drinks, tobacco, drugs, or excessive tea or coffee. Children 6–11 months Artificial sweeteners (saccharin, aspartame, sucralose, stevia, or other) should not be used in children’s foods under 2 years of age directly or in preparations or commercial products labeled “light” or “diet” or other similar ones. Powdered drinks, juices or nectars with sugar, soft drinks, and in general any sugary drink or with artificial sweeteners, are not recommended, nor necessary.
Colombia ^UMI^ 2014	*To maintain a healthy weight, reduce the consumption of packaged products, fast foods, soft drinks, and sweetened drinks*. Pregnant women Avoid the consumption of products like soft drinks, energy drinks, and sugary drinks that favor the onset of diabetes, being overweight, and obesity. Lactating mothers Do not consume alcoholic beverages or energy drinks.
Costa Rica ^UMI^ 2010	Decrease the amount of sugar that is used to sweeten your drinks. Choose juices or drinks that are 100% natural, without sugar. Avoid the consumption of pastries, cookies, condensed milk, dulce de leche, soft drinks, sweets, chocolates, ice creams, jellies, and jams. In addition to water, you can consume other liquids like juices, tea, broths, and soups.
Cuba ^UMI^ 2009	Decrease the consumption of all types of sweets (homemade, industrial, candies, jams and others), as well as sweetened drinks.
Dominica ^UMI^ 2007	*Choose less sweet foods and drinks*.
Dominican Republic ^UMI^ 2009	Prefer medium and low energy foods to achieve energy balance. Foods high in energy include: fritters, desserts, oils, margarine, butter, lard, mayonnaise, picaderas, pork rinds, bacon, fast food, snacks, dressings, stuffed crackers, sausages, pastries, and carbonated and alcoholic beverages.
El Salvador ^LMI^ 2012	*Avoid eating sugary foods and drinks, chips, sausages, sweets, highly processed foods, and canned foods*. These foods have delicious taste and appetizing appearance, but they provide large amounts of saturated fat and calories. Therefore, the consequences of frequent consumption are: becoming overweight and obesity, the appearance of diseases of the heart, diabetes mellitus, hypertension, tumors and cancers, and dental caries.
Grenada ^UMI^ 2016	*Choose to use less sweet foods and drinks*.
Guatemala ^LMI^ 2012	Avoid the consumption of carbonated water, drinks energizers, bottled drinks with artificial flavors, packaged juices, etc., because they contain excess sugar, preservatives, and dyes that are harmful to health. Coffee consumption is not recommended as a substitute for water. Coffee stimulates acidity or secretion of gastric acids and produces discomfort in cases of diseases of the digestive system. The effect of tea, especially black tea, is similar.
Honduras ^LMI^ 2013	Do not substitute water for coffee or tea, as they can cause acidity and other problems in the digestive system. Avoid the consumption of soft drinks, energy drinks, bottled beverages, packaged juices, juices and natural soft drinks, etc., since they generally contain large amounts of sugar, dyes, and preservatives that are harmful to health.
Jamaica ^UMI^ 2015	*Reduce intake of sugary foods and drinks*. These include honey, syrup, jam, sweetened carbonated beverages, condensed milk, sweet snacks, and desserts. Benefits: reduces risk of becoming overweight/obesity, hypertension, diabetes, heart diseases, and other chronic illness.
Mexico ^UMI^ 2015	*Drink plenty of plain water. Drink plain aguas frescas or flavored water without added sugar instead of sweetened drinks such as soft drinks, juices, and aguas frescas*. Decrease the consumption of energy-containing beverages such as soft drinks, nectars, and sweetened drinks with fruit flavors. Mexico has one of the highest consumption of sugary drinks in the world for all age groups from 1 year old. The types of beverages that contribute the greatest to energy in the population are: soft drinks (carbonated and not carbonated); drinks made with fruit juices (with or without sugar), which are taken as natural juices; fresh waters; juices made with 100% fruit; and flavored milks. Beverage classes according to their energy content, nutritional value, and risks to health on a scale that classifies drinks from the most (level 1) to the least (level 5) healthy. Level 1: Drinking water; Jamaican water without sugar (six to eight glasses of water per day) Level 2: Low-fat milk (1%) or fat-free and sugar-free (max two glasses a day) Level 3: Coffee, tea, and fruit water without sugar (maximum four cups a day) Level 4: Drinks with high calorific value and limited health benefits (fruit juices, whole milk, fruit smoothies with sugar or honey, and sports drinks) (maximum 1/2 cup per day) Level 5: Drinks with sugar and low nutrient content
Panama ^UMI^ 2013	*Avoid sodas, iced tea, and sugary drinks. Prefer natural juices without sugar*. The frequent or daily consumption of sodas or sugary drinks leads to the appearance of obesity, diabetes, hypertension, and high cholesterol, as well as cardiovascular and renal diseases. Avoid all colors and flavors of sodas, iced teas of any flavor, and sugary drinks with colorants, packaged, or with powder.
Paraguay ^UMI^ 2015	*Drink less carbonated beverages and artificial juices because they damage your health*. Consume less sweets, sodas, and sweetened drinks to stay healthy. Foods high in simple sugars contribute empty calories without special nutrients, and so excessive consumption can harm your health when these foods rich in sugars are eaten in excess, they are accumulated in the body in the form of fat, which is considered a risk factor of obesity, cardiovascular disease (heart and arteries), diabetes, dental cavities, and others.
Saint Kitts and Nevis ^HI^ 2010	*Limit the use of foods and drinks with added salt and sugar*.
Saint Lucia ^UMI^ 2007	*Choose fewer beverages and foods preserved or prepared with added sugar*.
Saint Vincent and the Grenadines ^UMI^ 2006	*Reduce the intake of sugar: use less sugar, sweet foods, and drinks*.
Uruguay ^HI^ 2016	*Base your diet on natural foods, and avoid the regular consumption of ultra-processed products with excessive contents of fat, sugar, and salt*. *Prefer water to other beverages. Limit sodas, artificial juices, and flavored waters*.
Venezuela ^UMI^ 1991	Soft drinks and other sugary drinks only provide calories. In contrast, natural fruit juices prepared at home provide an addition of calories, vitamins, and minerals
**NORTH AMERICA**	
Canada ^HI^ 2007	Limit foods and beverages high in calories, fat, sugar or salt (sodium) [many listed, i.e., soft drinks, sports and energy drinks, and sweetened hot or cold drinks, hot chocolate, specialty coffee]. Have a glass of low fat milk rather than pop or fruit drinks. Use low-fat evaporated milk instead of cream or coffee whitener in coffee or tea. Soft drinks, sports drinks, energy drinks, and alcoholic beverages can add a significant number of calories to the diet. These drinks may also contain caffeine or sodium. Health Canada: https://www.canada.ca/en/health-canada/services/food-nutrition/food-safety/food-additives/caffeine-foods/foods.html A review (Nawrot et al, Food Additives and Contaminants, 2003) undertaken by Health Canada scientists has considered the numerous studies dealing with caffeine and its potential health effects. It has re-confirmed that for the average adult, moderate daily caffeine intake at dose levels of 400 mg/day is not associated with any adverse effects. Data has shown, however, that women of childbearing age and children may be at greater risk from caffeine. Recommended max caffeine intake levels. 4–6 years: 45 mg/day 7–9 years: 62.5 mg/day 10–12 years: 85 mg/day Women planning to become pregnant, pregnant women, and breast feeding mothers: 300 mg/day Suggestions for adolescents 13+ years: 2.5 mg/kg body weight.
United States ^HI^ 2016	*Limit calories from added sugars and saturated fats and reduce sodium intake*. When choosing beverages, both the calories and nutrients they may provide are important considerations. Beverages that are calorie-free, especially water, or that contribute beneficial nutrients, such as fat-free and low-fat milk and 100% juice, should be the primary beverages consumed. Milk and 100% fruit juice should be consumed within recommended food group amounts and calorie limits. Sugar-sweetened beverages, such as soft drinks, sports drinks, and fruit drinks that are less than 100% juice, can contribute excess calories while providing few or no key nutrients. If they are consumed, amounts should be within overall calorie limits and limits for calories from added sugars. Caffeine is not a nutrient; it is a dietary component that functions in the body as a stimulant. Caffeine occurs naturally in plants (e.g., coffee beans, tea leaves, cocoa beans, and kola nuts). It is also added to foods and beverages (e.g., caffeinated soda and energy drinks). Moderate coffee consumption (three to five 8-oz cups/day or providing up to 400 mg/day of caffeine) can be incorporated into healthy eating patterns. This guidance on coffee is informed by strong and consistent evidence showing that, in healthy adults, moderate coffee consumption is not associated with an increased risk of major chronic diseases (e.g., cancer) or premature death, especially from CVD. Individuals who do not consume caffeinated coffee or other caffeinated beverages are not encouraged to incorporate them into their eating pattern. Limited and mixed evidence is available from randomized controlled trials examining the relationship between those energy drinks which have high caffeine content and cardiovascular risk factors and other health outcomes. Caffeinated beverages, such as some sodas or energy drinks, may include calories from added sugars, and although coffee itself has minimal calories, coffee beverages often contain added calories from cream, whole or 2% milk, creamer, and added sugars, which should be limited. The same considerations apply to calories added to tea or other similar beverages. Those who choose to drink alcohol should be cautious about mixing caffeine and alcohol together or consuming them at the same time. Women who are capable of becoming pregnant or who are trying to, or who are pregnant, and those who are breastfeeding should consult their health care providers for advice concerning caffeine consumption.

HI: high-income; LI: low-income; LMI: lower-middle-income; UMI: upper-middle-income; CVD: cardiovascular disease.

## Supplementary Materials

The following are available online at http://www.mdpi.com/2072-6643/10/11/1772/s1, Figure S1: Data Collection Strategy for Dietary Caffeine Guidelines; Table S1: Country Specific Guidelines Pertaining to Dietary Caffeine.
