# Supplementary material for: Caffeine in the Diet: Country-Level Consumption and Guidelines
Source: Nutrients. 2018 Nov 15;10(11):1772. doi: 10.3390/nu10111772 (PMC6266969; doi:10.3390/nu10111772)

Caffeine in the diet: Country-level consumption and guidelines

Celine Marie Reyes and Marilyn C Cornelis

Supplementary Information

Table S1. Country Specific Guidelines Pertaining to Dietary Caffeine

Country	Date	Intended audience
Benin[LI]	2015	Healthy population 2+ years, primarily in urban and semi-urban settings. Specific guidelines for children, pregnant/nursing women, and other age groups.
Nigeria[LMI]	2001	Healthy population. Specific guidelines for children, pregnant/nursing women, and other age groups.
Seychelles[HI]	2006	Healthy population 2+ years. Specific guidelines for children and pregnant/nursing women.
Sierra Leona[LI]	2016	Healthy population. Specific guidelines for children, pregnant/nursing women, and other age groups.
South Africa[UMI]	2012	General population 5+ years.<br>Specific guidelines proposed for children 0–5 years and pregnant/nursing women.
Afghanistan[LI]	2015	General population. Specific guidelines for children, pregnant/nursing women, and other age groups.
Australia[HI]	2013	Healthy population 2+ years. Specific guidelines for children, pregnant/nursing women, and other age groups.
Bangladesh[LMI]	2013	General population. Specific guidelines for children <2 years and pregnant/nursing women.
China[UMI]	2016	Healthy population 2+ years. Specific guidelines for children, pregnant/nursing women, and other age groups.
Fiji[UMI]	2013	Healthy population. Specific guidelines for children and pregnant/nursing women.
India[LMI]	2011	General population. Specific guidelines for children, pregnant/nursing women, and other age groups.
Indonesia[LMI]	2014	General population. Specific guidelines for children, pregnant/nursing women, and other age groups.
Japan[HI]	2010	Healthy population. Specific guidelines for children.
Malaysia[UMI]	2010	General population 2+ years.
Nepal[LI]	2012	General population. Specific guidelines for children and pregnant/nursing women.
New Zealand[HI]	2008<br>2012<br>2008<br>2013	Infants and toddlers (0-2 years)<br>Children and young people (2-18 years)<br>Pregnant/nursing women<br>Older people
Republic of Korea[HI]	2010	Specific guidelines for children, pregnant/nursing women, and other age groups.
Sri Lanka[LMI]	2011	General population. Specific guidelines for children, pregnant/nursing women, and other age groups.
Thailand[UMI]	2008-10	General population 6+ years. Specific guidelines for children, pregnant/nursing women, and other age groups.
Vietnam[LMI]	2013	General population. Specific guidelines for children and pregnant/nursing women.
Iran[UMI]	2015	General population 2+ years.
Lebanon[UMI]	2013	Adult population. Specific guidelines for children and pregnant/nursing women.
Qatar[HI]	2015	Healthy population 2+ years. Specific guidelines for children and pregnant/nursing women.
Albania[UMI]	2008	General population. Specific guidelines for children, pregnant/nursing women, and other age groups.

Austria[HI]	2010	General population. Specific guidelines for children, pregnant/nursing women, and other age groups.
Belgium[HI]	2005	General population. Specific guidelines for children, pregnant/nursing women, and other age groups.
Bulgaria[UMI]	2006	Adult population. Specific guidelines for children and pregnant/nursing women.
Croatia[HI]	2002	Specific guidelines for pregnant/nursing women and other age groups.
Cyprus[HI]	2007	General population. Specific guidelines for age groups.
Denmark[HI]	2013	Healthy population 3+ years. Specific guidelines for children and pregnant/nursing women.
Estonia[HI]	2006	General adult population. Specific guidelines for children, pregnant/nursing women, and other age groups.
Finland[HI]	2014	Healthy population. Specific guidelines for children, pregnant/nursing women, and other age groups.
France[HI]	2011	General population. Specific guidelines for children, pregnant/nursing women, and other age groups.
Georgia[UMI]	2005	General population. Specific guidelines for children, pregnant/nursing women, and other age groups.
Germany[HI]	2013	Healthy population.
Greece[HI]	1999	Adult population.
Hungary[HI]	2004<br>2016	Healthy adult population. Specific guidelines for pregnant/nursing women.
Ireland[HI]	2012	General population 5+ years. Specific guidelines for children and pregnant/nursing women.
Israel[HI]	2008	General population.
Italy[HI]	2003	Healthy population. Specific guidelines for children, pregnant/nursing women, and other age groups.
Latvia[HI]	2008<br>2003<br>2007	Adults (and pregnant/nursing women)<br>Children<br>Elderly
Malta[HI]	2016	General adult population. Specific guidelines for children and pregnant/nursing women.
Netherlands[HI]	2016	General population. Specific guidelines for children and pregnant/nursing women.
Norway[HI]	2014	General population. Specific guidelines for children, pregnant/nursing women, and other age groups.
Poland[HI]	2010	General adult population. Specific guidelines for children, pregnant/nursing women, and other age groups.
Portugal[HI]	2003	Healthy population. Specific guidelines for children and pregnant/nursing women.
Romania[UMI]	2006	Healthy population. Specific guidelines for children and pregnant/nursing women.
Slovenia[HI]	2011	Healthy population 2+ years.
Spain[HI]	2008	General population. Specific guidelines for children, pregnant/nursing women, and other age groups.
Sweden[HI]	2015	Healthy population 2+ years. Specific guidelines for children, pregnant/nursing women, and other age groups.
Switzerland[HI]	2011	Healthy adult population. Specific guidelines for age groups.
Turkey[UMI]	2006	General population. Specific guidelines for children and pregnant/nursing women.
United Kingdom[HI]	2016	General population 2+ years. Specific guidelines for children and pregnant/nursing women.

Antigua and Barbuda[HI]	2013	General population 2+ years.
Argentina[UMI]	2015	General population 2+ years. Specific guidelines for children and pregnant/nursing women.
Bahamas[HI]	2002	Healthy population 2+ years. Specific guidelines for children and pregnant/nursing women.
Barbados[HI]	2017	General population 2+ years.
Belize[HI]	2012	Healthy population 2+ years.
Bolivia (Plurinational State of)[LMI]	2013	Healthy population 2+ years. Specific guidelines for children, pregnant/nursing women, and other age groups.
Brazil[UMI]	2014	General population 2+ years.<br>Specific guidelines for children <2 years and pregnant/nursing women.
Chile[HI]	2013	General population 2+ years. Specific guidelines for children, pregnant/nursing women, and other age groups.
Colombia[UMI]	2014	Healthy population 2+ years. Specific guidelines for children, pregnant/nursing women, and other age groups.
Costa Rica[UMI]	2010	Healthy population 2+ years.
Cuba[UMI]	2009	General population 2+ years. Specific guidelines for children <2 years and pregnant/nursing women.
Dominica[UMI]	2007	Healthy population 2+ years.
Dominican Republic[UMI]	2009	Healthy population 2+ years. Specific guidelines for children <2 years and pregnant/nursing women.
El Salvador[LMI]	2012	Healthy population 2+ years. Specific guidelines for children and pregnant/nursing women.
Grenada[UMI]	2016	Healthy population 2+ years.
Guatemala[LMI]	2012	General population 2+ years Specific guidelines for children <2 years and pregnant/nursing women.
Honduras[LMI]	2013	Healthy population 2+ years. Specific guidelines for children and pregnant/nursing women.
Jamaica[UMI]	2015	General population 2+ years.
Mexico[UMI]	2015	General population. Specific guidelines for children, pregnant/nursing women, and other age groups.
Panama[UMI]	2013	Healthy population 2+ years. Specific guidelines for children and pregnant/nursing women.
Paraguay[UMI]	2015	General population 2+ years. Specific guidelines for children <2 years and pregnant/nursing women.
Saint Kitts and Nevis[HI]	2010	Healthy population 2+ years.
Saint Lucia[UMI]	2007	Healthy population 2+ years.
Saint Vincent and the Grenadines[UMI]	2006	Healthy population 2+ years.
Uruguay[HI]	2016	General population 2+ years. Specific guidelines for children and pregnant/nursing women.
Venezuela[UMI]	1991	General population 2+ years. Specific guidelines for children and pregnant/nursing women.
Canada[HI]	2007	General population 2+ years. Specific guidelines for children and pregnant/nursing women.
United States[HI]	2016	General population 2+ years. Specific guidelines for children and pregnant/nursing women.

HI: high-income; LI: low-income; LMI: lower-middle-income; UMI: upper-middle-income

Figure S1. Data Collection Strategy for Dietary Caffeine Guidelines

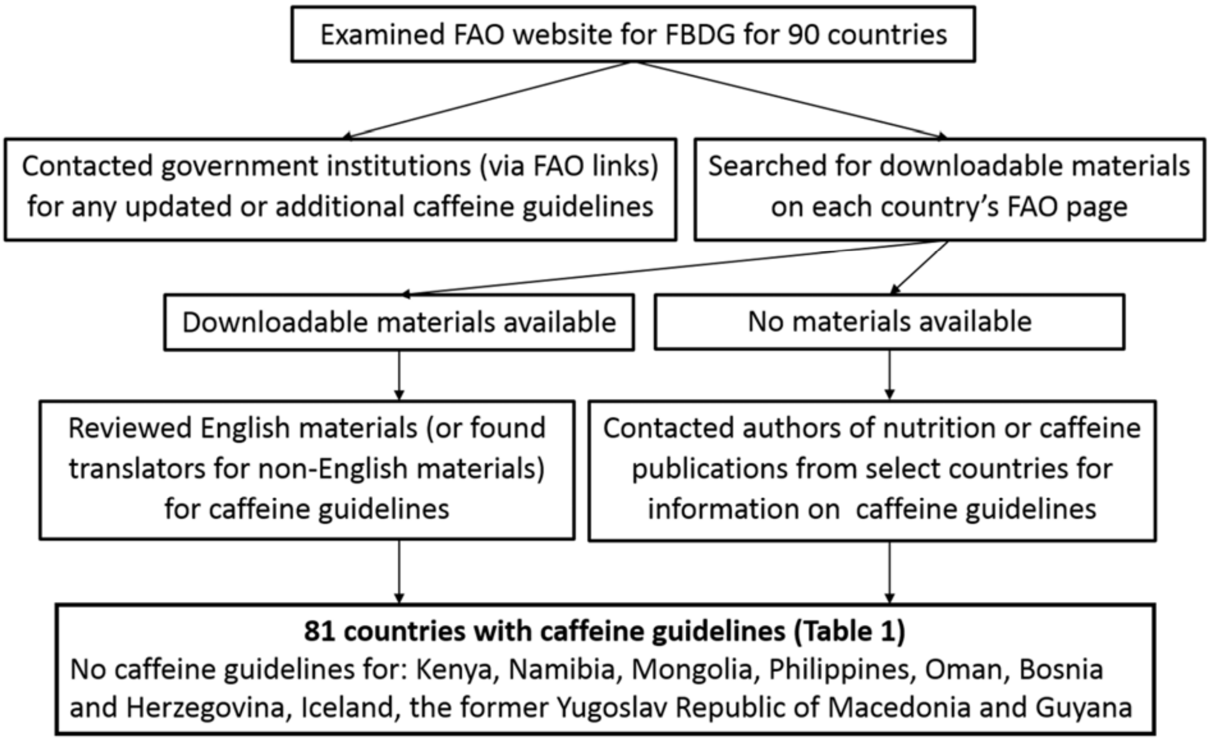

Supplement: Supplementary file 1 [file nutrients-10-01772-s001.pdf]
